# Sustainable carbon dots from *Borreria hispida*: enhanced colorimetric sensing of Fe^3+^ ions and biological applications in live cell imaging†

**DOI:** 10.1039/d4ra01686f

**Published:** 2024-05-30

**Authors:** Shanmuga Priya S, Suseem SR

**Affiliations:** a Department of Chemistry, School of Advanced Sciences, Vellore Institute of Technology Vellore 632014 Tami Nadu India srsuseem@vit.ac.in Shanmugapriyachemist@gmail.com

## Abstract

This study presents the synthesis of advanced nanomaterials derived from the hedge-grown herbal plant, *Borreria hispida*, and explores their environmental and biological applications. Using a one-step hydrothermal synthesis method, carbon dots derived from *Borreria hispida* (BHCD) were fabricated and thoroughly characterized through XRD, TEM, FTIR, CHNS, UV-visible, and PL spectroscopy analyses. Under UV illumination, these plant-based carbon dots demonstrated exceptional water solubility, notable photo stability, and a high quantum yield of 40.8%. The average particle size of BHCD was absorbed around 0.5 to 3.5 nm, contributing to superior selectivity and sensitivity in detecting Fe^3+^ ions, with a limit of detection of 1.2 × 10^−6^ M. Investigation into the sensing mechanism revealed a binding model wherein two carbon atom molecules bind to one Fe^3+^ atom in a 2 : 1 ratio for BHCDs and Fe^3+^ interactions. Additionally, the effectiveness of the developed fluorescent probe for Fe^3+^ detection was validated using real water samples from ponds and lakes, highlighting its potential for environmental monitoring applications. Furthermore, the biological effects of BHCD were evaluated through cytotoxic assays, demonstrating significant inhibitory effects on MCF7 breast cancer cell lines, with a maximum cell viability of 60%. This research underscores the multifaceted potential of BHCD in environmental monitoring and biomedical applications.

## Abstract 2

This study presents the synthesis of advanced nanomaterials derived from the hedge-grown herbal plant, *Borreria hispida*, and explores their environmental and biological applications.

## Graphical abstract

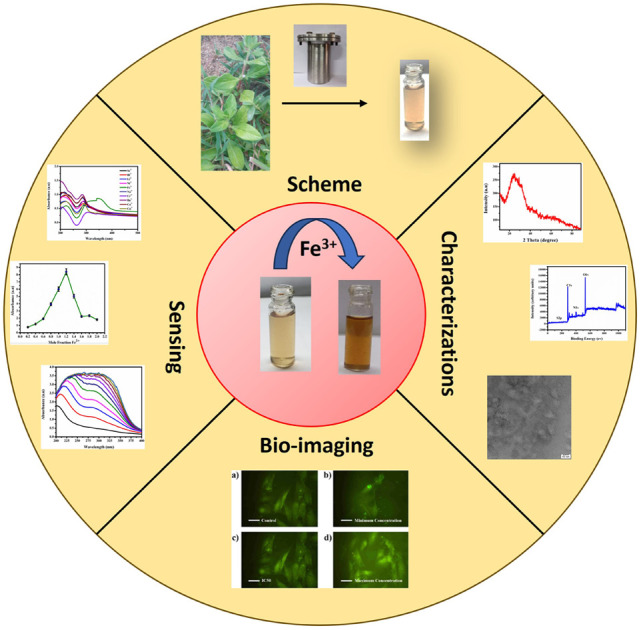

## Introduction

Iron, an essential element for life and classified as a transition metal, poses a significant threat to human health due to the increasing presence of Fe^3+^ ions in water resources. Elevated levels of Fe^3+^ in the human body have been linked to various health issues,^1,2^ including organ malfunctions such as those in the heart, pancreas, and liver. Multiple sources contribute to the rise of iron in drinking water, including the dissolution of ironstones, contamination from industrial wastewater, corrosion of water pipes, and the use of construction materials in drinking water resources.^3,4^ High concentrations of iron lead to water turbidity, while low concentrations promote bacterial growth, resulting in clogged water supply pipelines and unpleasant odours. Furthermore, a heightened iron concentration in water serves as an indicator for other heavy metals.^5,6^ The prevalence of iron in the environment and water resources surpasses that of other heavy metal ions like lead and mercury. Therefore, active, and precise monitoring of Fe^3+^ in drinking water is crucial.^7–9^

To achieve sustainable progress, it is crucial to recognize and eliminate harmful pollutants. However, the scientific community faces a significant challenge in integrating both aspects: identification and removal on a single platform.^10–12^ Promising technologies for detecting and eliminating toxic contaminants utilize fluorescence sensing and photo-degradation-based methods. These approaches are preferred for their numerous benefits, such as their ability to operate swiftly and straightforwardly, coupled with an energy-efficient mechanism.^13–15^

In the field of materials science, researchers have harnessed the enticing properties of nanomaterials like carbon dots (CDs), encompassing fluorescence, water solubility, exceptional thermos photo-stability, and either low or non-toxicity, along with the potential for scalable production.^16–18^ Through successful endeavours, they have synthesized quasi-spherical nano-sized CDs (<10 nm) using both top-down and bottom-up approaches. These methodologies yield CDs with a graphitic, crystalline, or amorphous sp^2^ hybridized carbon core and an oxidized carbon surface.^19–21^ In nanomaterial synthesis, top-down methodologies involve fragmenting carbon matter into carbon nanoparticles using diverse techniques such as arc discharge, laser ablation, electrochemistry, and wet oxidation. Conversely, the bottom-up approach entails transforming small carbon precursor molecules (such as citric acid, urea, or glucose) into carbon dots (CDs) of specific sizes.^20–22^ Researchers achieve this conversion using hydrothermal, ultrasonic, thermal decomposition, pyrolysis, carbonization, microwave synthesis, and solvothermal processes.^23–25^ Among the bottom-up approaches, pyrolysis is widely employed. In this process, organic material sourced from carbon undergoes a series of steps, including heating, dehydration, degradation, and carbonization under elevated temperatures in either vacuum or inert atmospheres.^26–28^ These CDs fall under sustainable nanotechnology, given the prevalent use of various plants, fruits, or bio-waste as carbon precursors.^29^ These CDs find vast and impactful applications across diverse fields such as bioimaging, cancer therapy, drug delivery, optoelectronic devices (including solar cells and light-emitting devices), catalysis, supercapacitors, agriculture, and optical sensors designed for detecting pollutants and heavy metals.^30^

To bridge the gap, we propose exploring the synthesis of carbon dots derived from hedge-grown plants. We will investigate these carbon dots, previously reported from edible plants like bananas and well-known herbal plants like Tulsi, for their environmental and biological applications, as work has yet to be reported on hedge-grown plants, particularly this plant (*Borreria hispida*). This study promises to contribute significantly to the field by expanding the path of carbon dots sources and unlocking new possibilities for their utilization in diverse technological and biomedical applications. By exploring this unreported approach in *Borreria hispida*, we aim to fill a critical void in the existing body of knowledge and pave the way for future advancements in carbon dot research. The novelty of this material lies not only in its botanical origin but also in its unexplored potential for specific applications such as metal sensing and bioimaging.

## Materials and methods

### Chemicals and reagents

The collected plant, *Borreria hispida*, was authenticated by the Botanical Survey of India, with the voucher specimen (BSI/SRC/5/23/2023-24/Tech/8). For the study, several substances were obtained, including dialysis membrane-60, 3-(4,5-dimethylthiazol-2-yl)-2,5-diphenyltetrazolium bromide (MTT), dimethyl sulfoxide (DMSO), 1X Antibiotic Antimycotic Solution, and 10% fetal bovine serum from Sigma Aldrich. Additionally, 1X Phosphate Buffered Saline (PBS) (Himedia, India), DAPI (4′,6-diamidino-2-phenylindole), an Optika IM-3FL4 fluorescent microscope (Optika, Germany), and cancer cell lines (MCF7) were acquired from the NCCS Pune.

### Characterization techniques

Various analytical methods were applied to the acquired BHCD. These encompassed Fourier Transform Infrared Spectroscopy (HITACHI), UV-visible absorbance spectroscopy (Jasco V-670) to establish the excitation range of the sample, and the examination of fluorescence properties using Fluorescence Spectroscopy (HITACHI F7000). Elemental analysis, determining the percentages of carbon, hydrogen, nitrogen, and sulfur in carbon dots, was conducted using a PerkinElmer −2400 CHNS/O series. The material's structure was elucidated through XRD analysis using a Bruker D8 Advance, while SEM analysis was carried out utilizing a Carl Zeiss EVO/18 Research microscope. High-Resolution Transmission Electron Microscopy (HR-TEM) was conducted using the FEI-Tecnai G2 20 Twin instrument, and X-ray Photoelectron Spectroscopy (XPS) was performed with the ULVAC-PHI Versa Probe 4. The synergy H1 microplate reader (Biotek) conducted the MTT assay, and fluorescence images were captured using the RTC-7 CON inverted fluorescence microscope.

### Sample preparation for sensing

We used distilled water to prepare a stock solution of BHCD (1 × 10^−2^ mol L^−1^). Fe^3+^ was generated by dissolving FeCl_3_ in water to achieve a concentration of 0.1 mol L^−1^. Additionally, various anion stock solutions (Se^2+^, Bi^2+^, Li^2+^, Ni^2+^, Fe^3+^, Na^2+^, Cr^2+^, Ba^2+^, Ca^2+^, Cu^2+^) were prepared using distilled water at a concentration of 0.1 mol L^−1^. These solutions were diluted to attain a uniform 1 × 10^−2^ mol L^−1^ concentration. All subsequent optical spectral characterization studies utilized these precisely calibrated stock solutions, ensuring the accuracy and reliability of our experimental setup. This preparation establishes a solid foundation for further analytical investigations.

## Experimental section

### Plant selection and collection

We selected *Borreria hispida* among the hedge-grown herbal plants for our work due to its remarkable therapeutic properties and reported pharmacological activities. The methanolic extract of *Borreria hispida* seeds demonstrated anticancer activity,^31^ while the leaves exhibited anti-inflammatory activity.^32^ Consequently, we decided to explore the novel application of *Borreria hispida*-derived carbon dots for metal sensing and cell imaging. In August, we collected the plant in Village C.N. Pattadai, Vellore, Tamil Nadu, India. The plant, fully matured and grown in loam soil, was shade-dried for two weeks, powdered using an electric grinder, and stored for further use.

### Synthesis of carbon dots (BHCD)

**Scheme 1:**
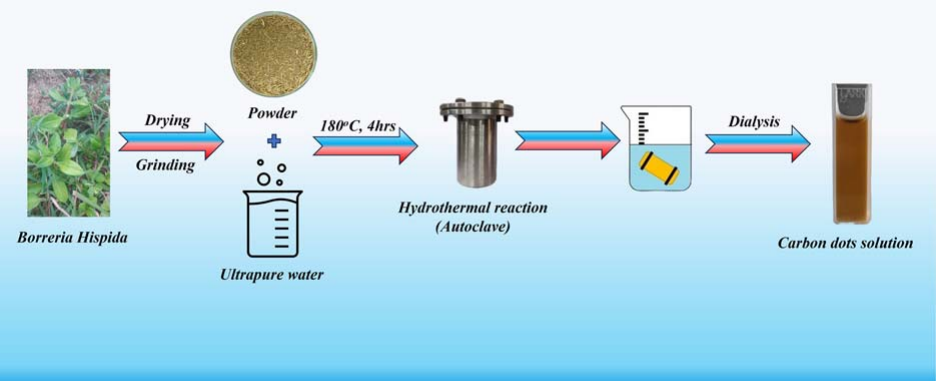
Synthetic route of *Borreria hispida* based carbon dots through hydrothermal reaction.

To synthesize carbon dots using the one-pot hydrothermal method with *Borreria hispida*, dissolve 5 g of powdered leaves of *Borreria hispida* in 125 mL of deionized water. Stir the mixture for 10 minutes and subject it to 10 minutes of sonication. Transfer the solution into a Teflon-lined autoclave and place it in a muffle furnace at 180 °C for 4 hours. Afterward, filter the solution using Whatman filter paper. Take the filtrate to separate large molecules through the dialysis method. Pack the solution into a membrane bag and allow it to contact Milli-Q water for purification over 14 hours. Finally, refrigerate the purified solution at 4 °C for future use. Scheme 1 explains this one-step synthesis using an herbal plant for producing sustainable carbon dots.

## Results and discussions

### Structural characterization

The utilization of *Borreria hispida*, identifiable by its white flowers, holds substantial promise as a natural source for producing carbon quantum dots (CQDs) owing to its abundant carbon, oxygen, and nitrogen elements across the entire plant structure. We employed a one-step hydrothermal synthesis method, yielding a CQD solution displaying a faint brown colour, indicative of the successful carbonization of the *Borreria hispid* plant material. Notably, the CQD solution showcased a measured pH of approximately 6, suggesting the presence of surface functional groups such as –OH and –COOH.

**Fig. 1:**
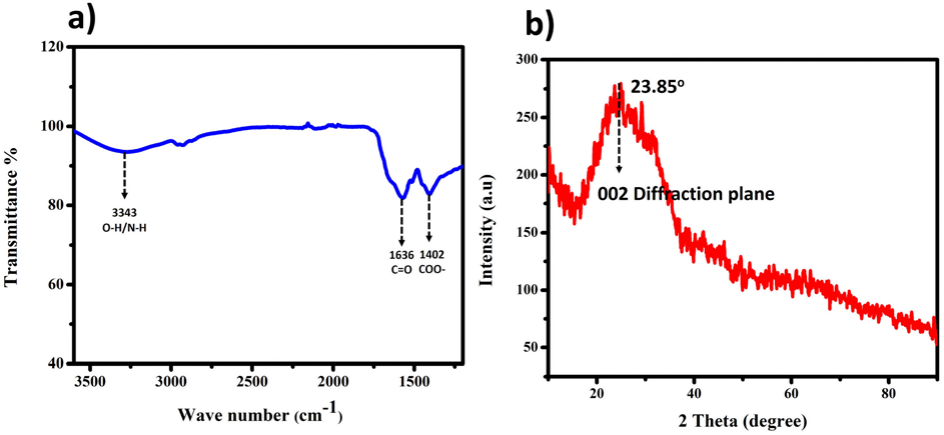
(a) FT-IR spectrum of BHCD. (b) Powder X-ray diffraction (PXRD) of BHCD.

The interpreted peaks from the Fourier Transform Infrared (FT-IR) spectra, illustrated in Fig. 1(a), are compelling evidence confirming the diverse chemical functionalities within the CQDs. Specifically, a medium peak observed around 3343 cm^−1^ implies the stretching of O–H and N–H bonds, indicating the presence of aliphatic primary amines. Additionally, the 1636 cm^−1^ and 1403 cm^−1^ peaks correspond to the C=O stretching and COO– stretching vibration.

To obtain X-ray diffraction (XRD) patterns for Thin-Layer Chromatography Liquid Crystal Displays (BHCDs), we applied a solution of BHCDs onto a pristine glass slide, left it to desiccate overnight at 50 °C, resulting in the formation of a thin film, as depicted in Fig. 1(b). We determined the interplanar distance (*d*-spacing) values for BHCDs using Bragg's equation, involving parameters such as the value of theta, which is 23.85, and the plane value of 002, consistent with that previously reported for CDs (position of the plane), *n* (a positive integer – in this case, 1), and *λ* (wavelength of the incident X-rays, where *λ* = 1.54 Å). Furthermore, we computed interatomic distances (a), with ‘*d*’ representing the interplanar distance and *h*, *k*, and *l* denoting the Miller indices.

**Fig. 2:**
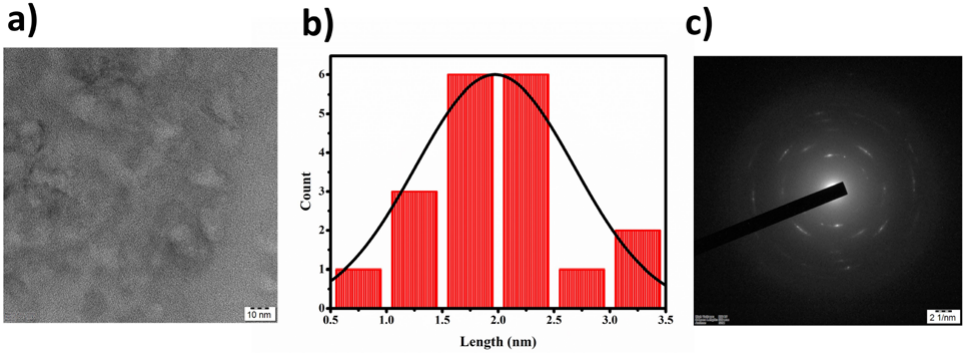
(a) HR-TEM images of BHCD. (b) Size distribution graph for BHCD. (c) SAEDP images of BHCD.

Through elemental analysis, we discerned the weight percentages of the constituent elements: carbon (41.57%), hydrogen (8.07%), nitrogen (11.75%), and sulfur (26.06%). Transmission Electron Microscopy (TEM) results, displayed in Fig. 2(a), revealed that the BHCDs exhibited a confined diameter range of 0.5–3.5 nm, showcasing an average crystallite size of 2 nm. The histogram in Fig. 2(b) visually portrays the size distribution of over 30 nanoparticles.

**Fig. 3:**
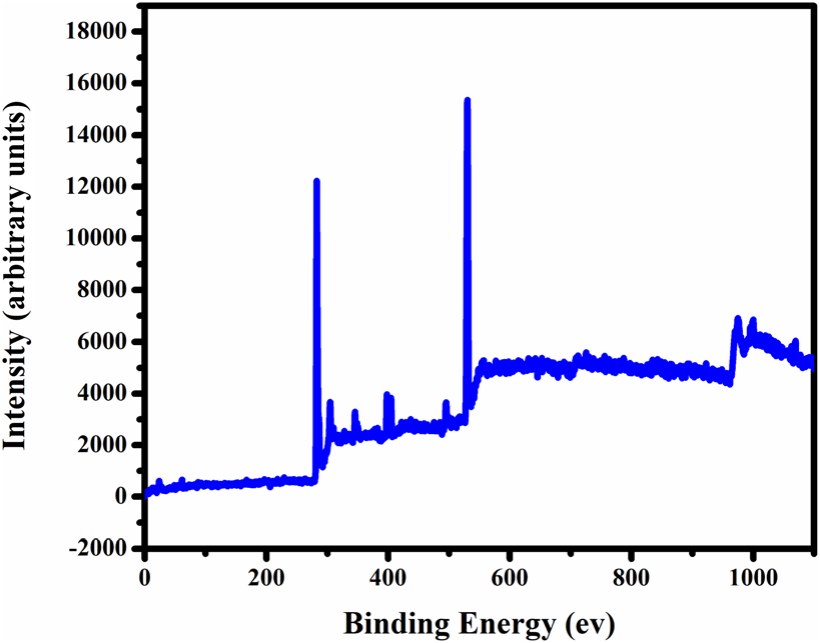
XPS spectrum of BHCD.

**Fig. 4:**
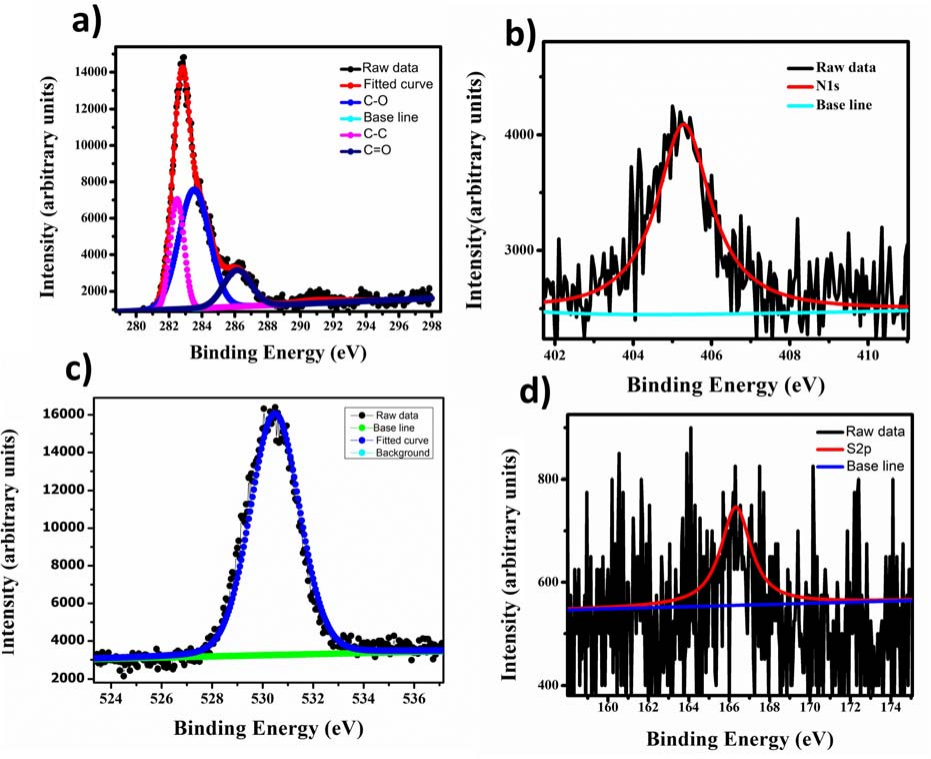
XPS spectrum in (a) C 1s region. (b) N 1s region. (c) O 1s region. (d) S 2p region.

In Fig. 3, we employed X-ray photoelectron spectroscopy (XPS) to analyse the synthesized carbon quantum dots (CQDs) for surface functional groups, chemical composition, and elemental states. The XPS full survey spectrum of CQDs showed distinct peaks at 529.3 eV, 284.4 eV, and 403.9 eV, corresponding to O 1s, C 1s, and 1N elements, respectively Fig. 4(a). We deconvoluted the high-resolution C 1s spectrum into three individual component peaks cantered at 282.4 eV, 283.5 eV, and 286.2 eV Fig. 4 (c). The deconvolution of the O 1s spectrum revealed three peaks at 530.4 eV, indicating oxygen functional groups on the surface of the carbon dots. Furthermore, the deconvolution of the N 1s signal in Fig. 4(b), observed at 405.3 eV, represented C–N, suggesting a low content of carbon-bonded nitrogen on the CQDs' surface. The high-resolution S 2p spectrum displayed three peaks at 166.3 eV, signifying the presence of C–S bonds within the carbon dots, as shown in Fig. 4(d). These results suggest that the synthesized CQDs are carbon-rich, and their functional groups predominantly consist of oxygen, nitrogen, and sulphur.

### Optical properties

**Fig. 5:**
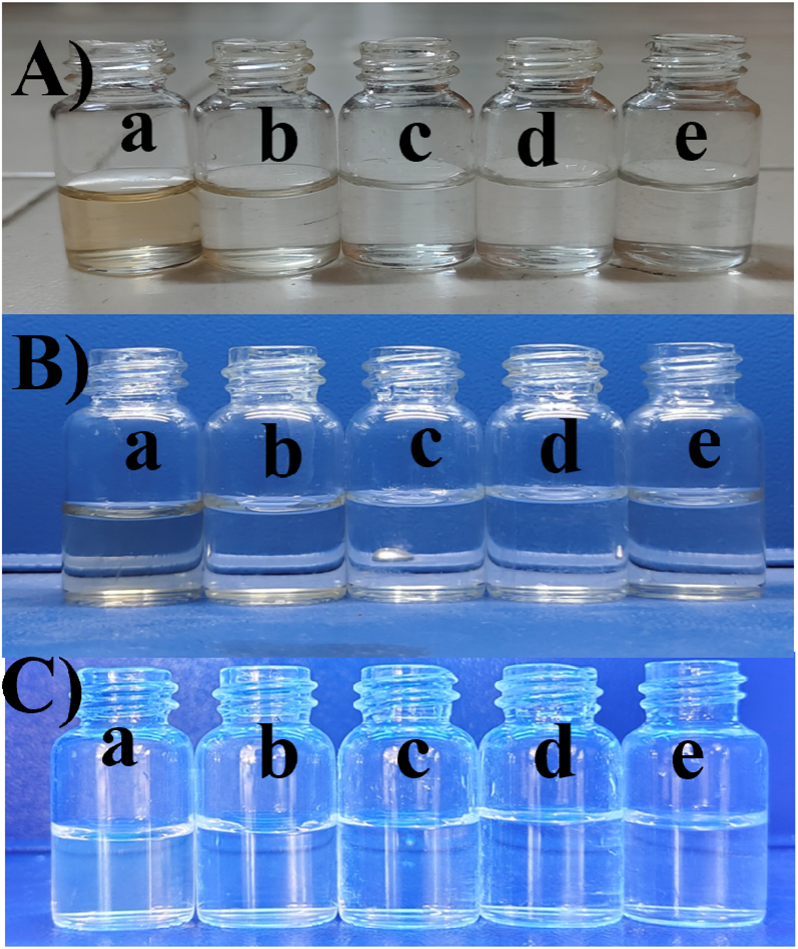
(A) Visible region (B) UV region, (C) FL region in the concentrations of (a) 1 M, (b) 0.1 M, (c) 0.01 M, (d) 0.001 M, and (e) 0.0001 M.

**Fig. 6:**
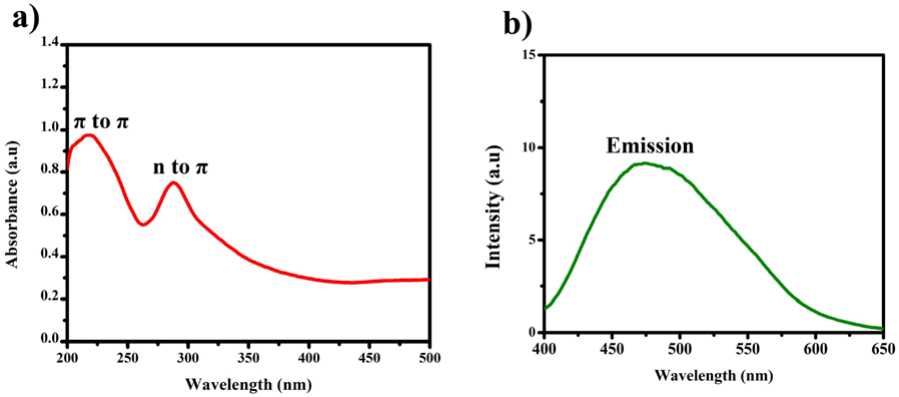
(a) UV-vis spectrum of BHCD (1 × 10^−2^ M) in water. (b) Fluorescence spectrum of BHCD (1 × 10^−2^ M) in water.

The UV-vis spectra revealed two distinct bands, each conveying significant transitions. At 210 nm, a distinct band indicated the π to π* transition, characterized by higher energy levels as shown in Fig. 6. Conversely, the band at 288 nm corresponded to the *n* to π* transition, similarly denoting higher energy involvement. The different concentrations of BHCD in different regions as shown in Fig. 5.

Intriguingly, the fluorescence spectra exhibited an emission peak at 475 nm. Notably, the naked eye immediately and noticeably perceived a change in colour upon observing the BHCDs compound, shifting from a light brown shade to a vibrant yellow shade.

**Fig. 7:**
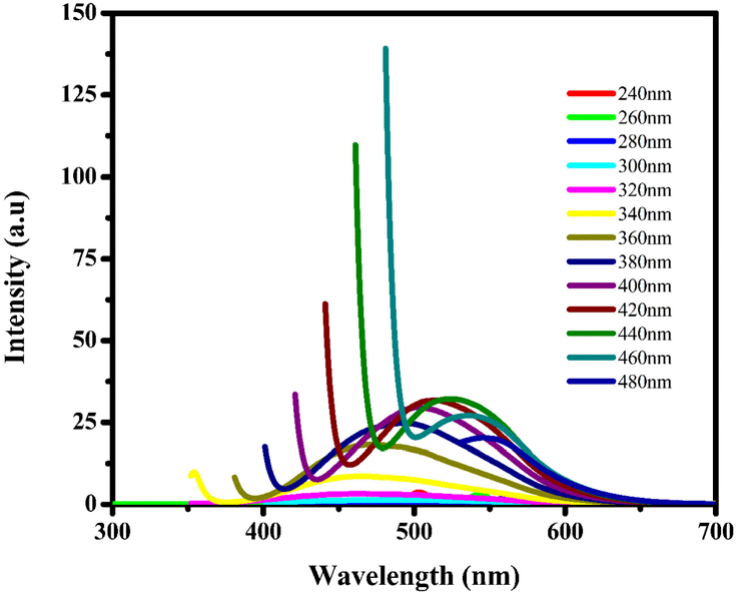
The fluorescence spectrum of BHCD in various excitation wavelengths.

Fluorescence spectroscopy analysis of the synthesized BHCDs is conducted under various wavelengths, as depicted in Fig. 7. Exciting CDs with shorter wavelengths increase their energy, enabling electrons to transition to higher vibrational states this reduction in emission energy results in an enhancement of Stokes shifts and a decrease in fluorescence intensity. The peak position shifts towards longer wavelengths as the excitation wavelength ranges from 240 to 480 nm.

### Metal ion sensing

#### Selectivity studies

**Fig. 8:**
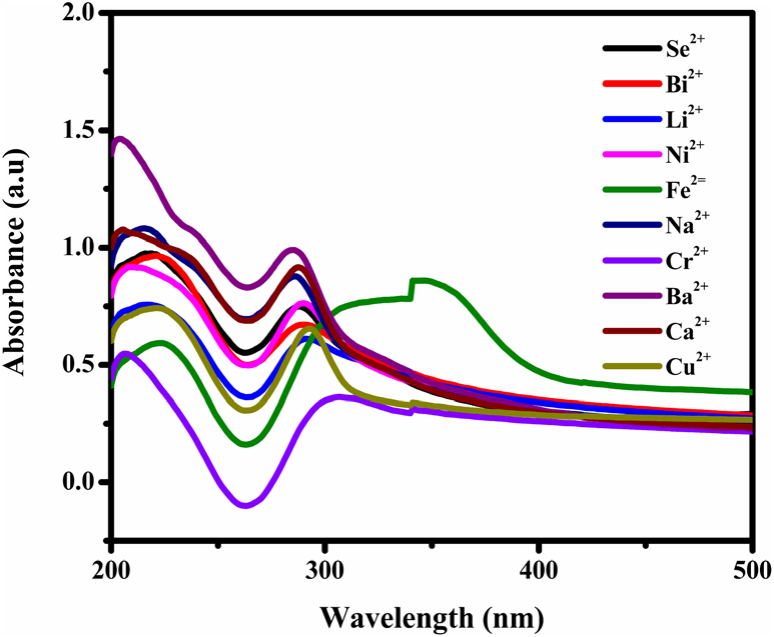
Selectivity studies of BHCD with various metal cations.

We utilized fluorescence spectroscopy for selectivity studies to evaluate the metal ion sensing capabilities of BHCDs. Aqueous solutions of various metal ions (Se^2+^, Bi^2+^, Li^2+^, Ni^2+^, Fe^3+^, Na^2+^, Cr^2+^, Ba^2+^, Ca^2+^, Cu^2+^) were prepared using their respective chemical salts. The objective was to investigate the selectivity of BHCDs concerning these metal ions. We incubated 0.2 mL of each metal ion for these experiments with a 1.8 mL solution of diluted BHCDs. This incubation lasted for 15 minutes, resulting in a final volume of 2 mL for each solution and a concentration of 200 μM for every metal ion. During this examination, Fig. 8 illustrates the absorbance, and the response of Fe^3+^ ions was particularly intriguing. Notably, the presence of Fe^3+^ induced a red shift in the fluorescence spectra, suggesting a distinctive response from BHCDs in the presence of this specific metal ion.

**Fig. 9:**
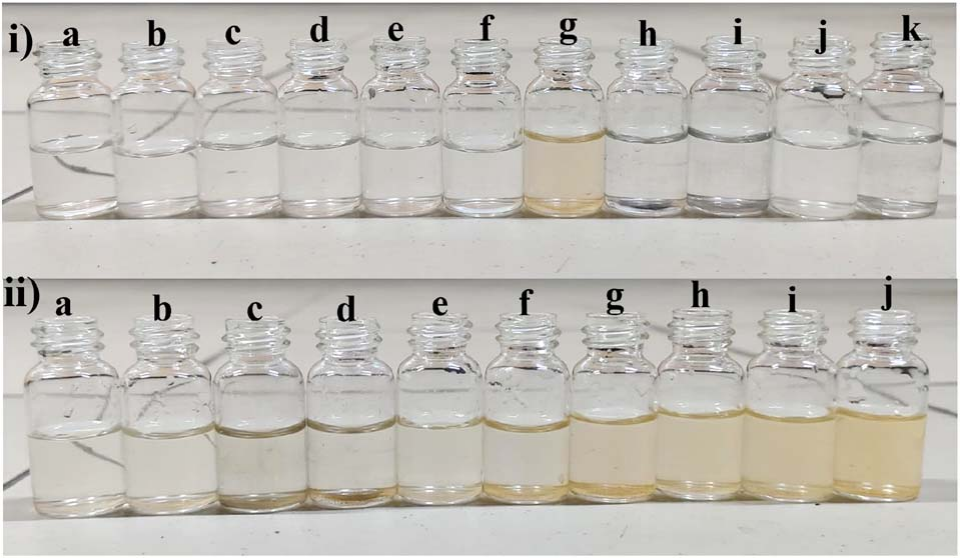
Colorimetric study of the BHCD (1 × 10^−2^ M) to the metals (i) (a) BHCD (b) Se^2+^, (c) Bi^2+^, (d) Li^2+^, (e) Ni^2+^, (f) Na^2+^, (g) Fe^3+^, (h) Fe^3+^, (i) Cr^2+^, (j) Ba^2+^, (k) Ca^2+^. (ii) (a) 0 μl, (b) 10 μl, (c) 20 μl, (d) 30 μl, (e) 40 μl, (f) 50 μl, (g) 60 μl, (h) 70 μl, (i) 80 μl, (j) 90 μl of Fe^3+^ in the concentrations of BHCD (1 × 10^−2^).

However, observations indicated that other metals did not induce significant changes in the absorbance spectra, implying a lack of substantial interaction or response from BHCDs in their presence. Fig. 9(i) and (ii) show noticeable colour changes in visible and UV light images upon detecting Fe^3+^ metal using BHCD and different concentrations. The observed changes demonstrate BHCD's sensitivity to Fe^3+^, with clear visible and UV spectra alterations. This visual representation emphasizes the effectiveness of BHCD as a Fe^3+^ detection sensor.

#### Sensitivity studies

**Fig. 10:**
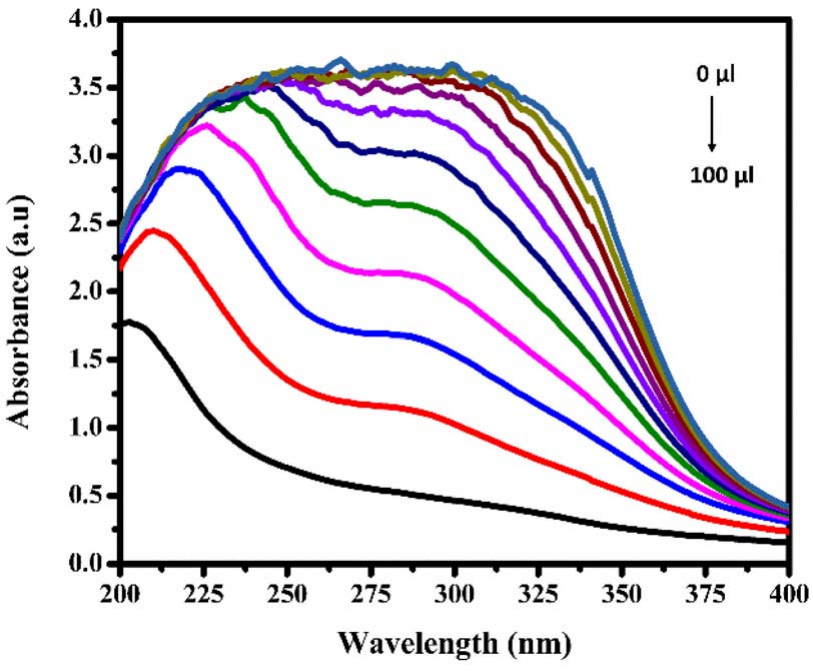
The UV-vis spectrum of BHCD towards various concentrations of Fe^3+^.

To confirm the interaction between Fe^3+^ ions and BHCDs, we investigated thoroughly to identify potential complex formation between the metal ions and the compound. Our focus cantered on examining the absorption spectra of BHCDs following the incremental addition of Fe^3+^ ions. The absorption band at 204 nm gradually decreased, while a new band emerged at 309 nm as shown in Fig. 10. This shift in the spectra strongly suggests a dynamic interaction between Fe^3+^ ions and BHCDs, with the diminishing intensity at 204 nm indicating a decline in the initial absorption band coinciding with the addition of Fe^3+^ ions.

Simultaneously, a distinct absorption band at 309 nm hints at forming a new entity resulting from the interaction between Fe^3+^ ions and BHCDs. This spectral evolution strongly suggests the potential formation of a compound due to the interaction between the metal ions and BHCDs, shedding light on the intricate nature of their interplay.

#### Binding studies

To investigate the binding affinity of Fe^3+^ ions with BHCDs, we conducted a comprehensive analysis using Job's plot, meticulously tracking absorbance changes across varying Fe^3+^ ion concentrations.

Furthermore, we determined the detection limit (LOD) and quantification limit (LOQ) using the standard formula *Kσ*/*s*, with *K* values of 3 for LOD and 10 for LOQ, as shown in Fig. 12(a) and (c). Consequently, we established the LOD and LOQ values for BHCDs as 1.2 × 10^−6^ M and 4.2 × 10^−7^ M, respectively.

**Fig. 11:**
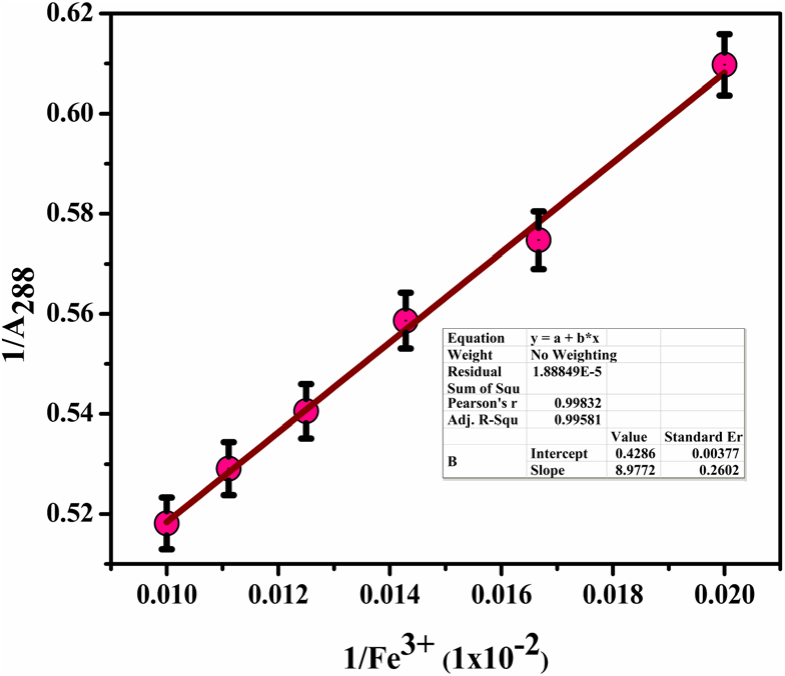
Benesi–Hildebrand plot of BHCD toward Fe^3+^.

By utilizing Job's plot, we successfully ascertained the stoichiometric ratio of BHCDs to Fe^3+^ ions as 2 : 1 as shown in Fig. 13. Moreover, we deduced the binding constant by assessing the ratio between the intercept and slope derived from observable colour changes upon the addition of Fe^3+^ ions to BHCDs under visible light conditions. The construction of the Bansi–Hildebrand plot, illustrated in Fig. 11 using the standard BH formula, clarifies this binding constant determination process. The quantification of the binding efficiency of Fe^3+^ ions with BHCDs was calculated as 4.77 × 10^2^ M^−1^, underscoring the robust binding affinity and selectivity of BHCDs, specifically for Fe^3+^ ions. This rigorous analysis illuminates the profound interaction and binding capabilities of BHCDs towards Fe^3+^ ions.

**Fig. 12:**
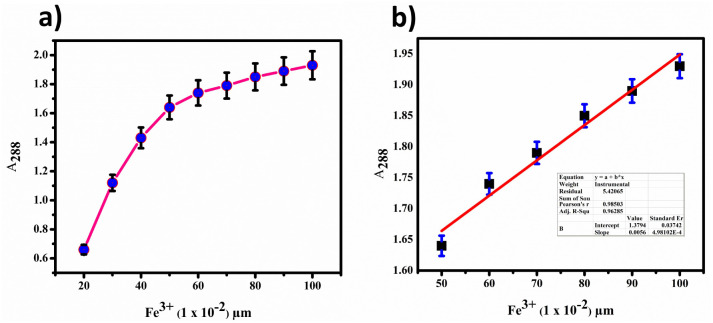
(a) Calibration plot for BHCD with Fe^3+^. (b) Calibration plot (linear fit) in the region of 50 to 100 μl.

**Fig. 13:**
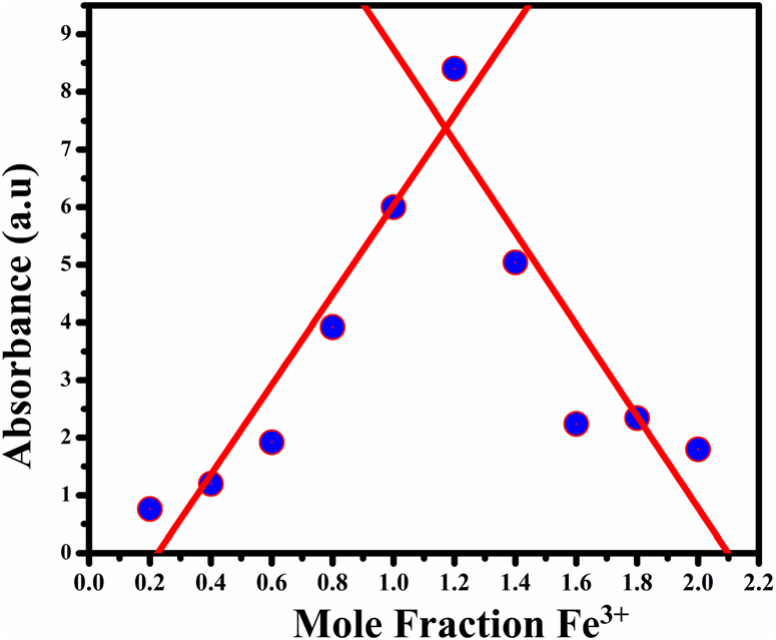
Jobs plot for BHCD in different mole fraction of Fe^3+^.

#### pH studies

**Fig. 14:**
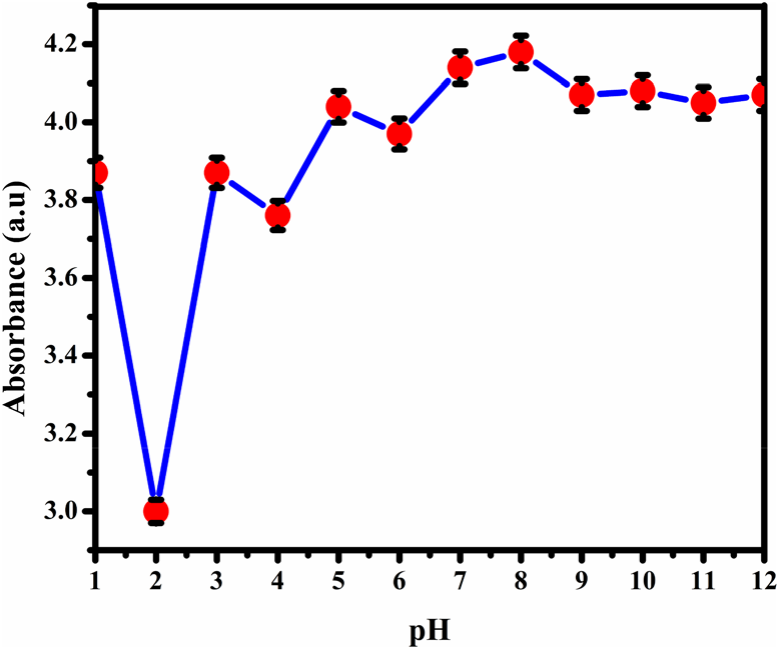
pH studies of BHCD with Fe^3+^ in different pH levels.

Nanoparticles significantly influence the stability and sensing capabilities, with the pH of the medium playing a crucial role. To enhance the sensing potential of BHCDs for Fe^3+^, we conducted experiments across a pH range of 1 to 12, controlling the pH using NaOH (0.1 M) for alkaline conditions and HCl (0.1 M) for acidic conditions. We recorded absorption spectra at room temperature, and Fig. 14 displays the absorbance *vs.* pH graph, demonstrating that BHCDs effectively recognize Fe^3+^ over a broad pH spectrum. We conducted further investigations to determine the binding stoichiometry of the BHCDs-iron complex.

#### Interference studies

**Fig. 15:**
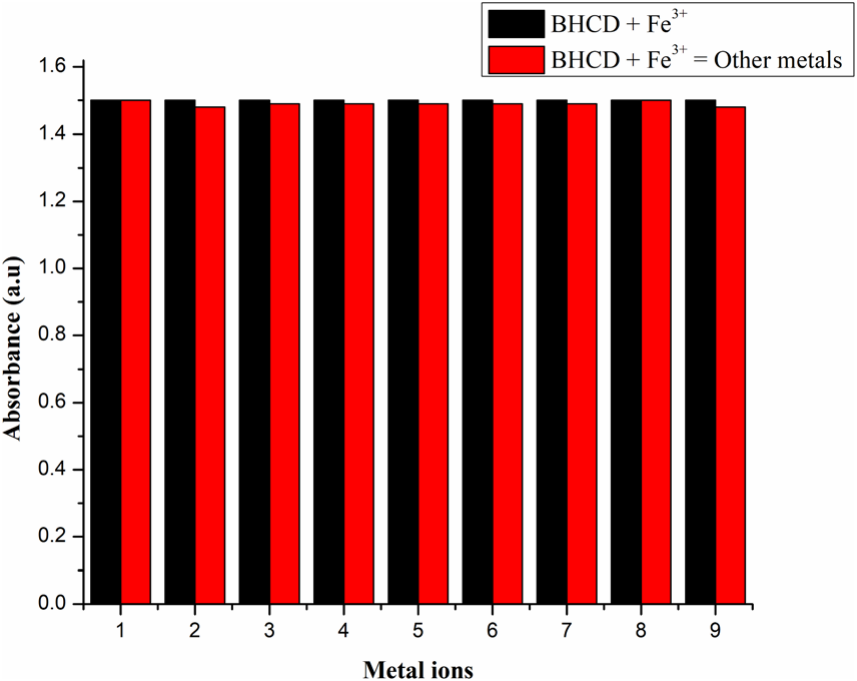
Interference studies BH + Fe^3+^ with various metal cations (1) Se^2+^, (2) Bi^2+^, (3) Li^2+^, (4) Ni^2+^, (5) Na^2+^, (6) Cr^2+^, (7) Ba^2+^, (8) Ca^2+^, (9) Cu^2+^.

We aimed to explore the interference potential of various anions on the accurate detection of iron metal by monitoring alterations in the absorption spectrum of the BHCD-Fe^3+^ solution. To assess this, we introduced 10 equivalents of diverse competing metals, encompassing Se^2+^, Bi^2+^, Li^2+^, Ni^2+^, Na^2+^, Cr^2+^, Ba^2+^, Ca^2+^, and Cu^2+^ ions. Interestingly as shown in Fig. 15, our observations revealed no discernible shifts in spectral changes or deviations in absorption maxima within the BHCD-Fe^3+^ solution, particularly at 345 nm. This recent project signifies the development of a sensing probe, displaying a significant leap forward compared to previously documented chemical-based probes. This exploration highlights the robustness and specificity of the BHCD-Fe^3+^ system as a potential sensing tool, offering enhanced accuracy in hypochlorite detection and setting a new benchmark in sensing technology.

#### Mechanism of Fe^3+^ sensing by BHCD

FT-IR spectroscopy revealed the presence of functional groups in the carbon dots. The C=O and COO– groups were confirmed at peaks around 1636 cm^−1^ and 1403 cm^−1^, respectively, while the O–H and N–H groups were identified at a peak around 3343 cm^−1^, suggesting the existence of secondary amines. In XPS analysis, the aldehyde (C–OH) group was identified at a binding energy of 286.2 eV in the C 1s spectrum. The ketone (C=O) and aldehyde (C–OH) groups were also observed at binding energies of 531.2 eV and 523.3 eV, respectively. The presence of the secondary amine (N–H) group was confirmed at a binding energy of 401.2 eV. Carbon, nitrogen, and oxygen were detected in the carbon dots, indicating the presence of functional groups such as OH, carbonyl (aldehyde, ketone), and amine. All these functional groups contain lone pairs that can donate electrons to the metal's empty d orbitals, forming coordinate bonds. This interaction results in changes in absorbance, providing insight into the mechanism through which carbon dots detect Fe^3+^.

#### Real-time water analysis

We evaluated the practical utility of BHCDs in detecting specific metal ions using tap water samples collected from our laboratory and pond and lake water obtained from Jambukulam, Vellore, spiked with a known concentration (50 μM) of Fe^3+^ ions. We filtered the water samples using 20 nm filter paper to remove impurities. Subsequently, we introduced varying concentrations of Fe^3+^, ranging from 0 to 20 μM, into the filtered samples. We measured the emission intensity at 475 nm during excitation to determine the Fe^3+^ concentration as shown in Fig. S2.† A standard UV-vis spectrum was a reference to validate the Fe^3+^ concentration in actual water samples. By comparing the emission intensities obtained from the water samples with the calibration curve, we estimated the corresponding Fe^3+^ concentrations. The comprehensive analysis of diverse water samples illustrated the accurate detection of the target compound. Importantly, we achieved recovery rates ranging from 98.6% to 104.3% using the BHCDs probe for Fe^3+^ detection, robustly confirming the efficiency of the BHCDs probe in the precise and quantitative detection of Fe^3+^ in real-world water samples.

### Bio imaging

**Fig. 16:**
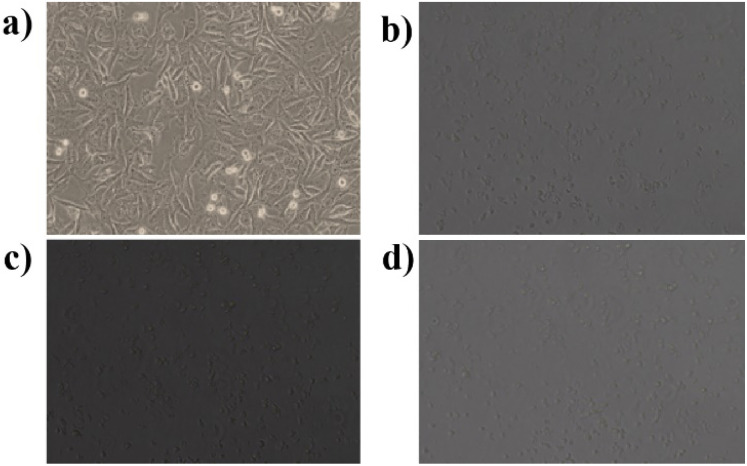
*In vitro* confocal microscopic imaging of MCF7 cells (a) control (b) 62.25 μg mL^−1^ (c) 125 μg mL^−1^ (d) 250 μg mL^−1^.

**Fig. 17:**
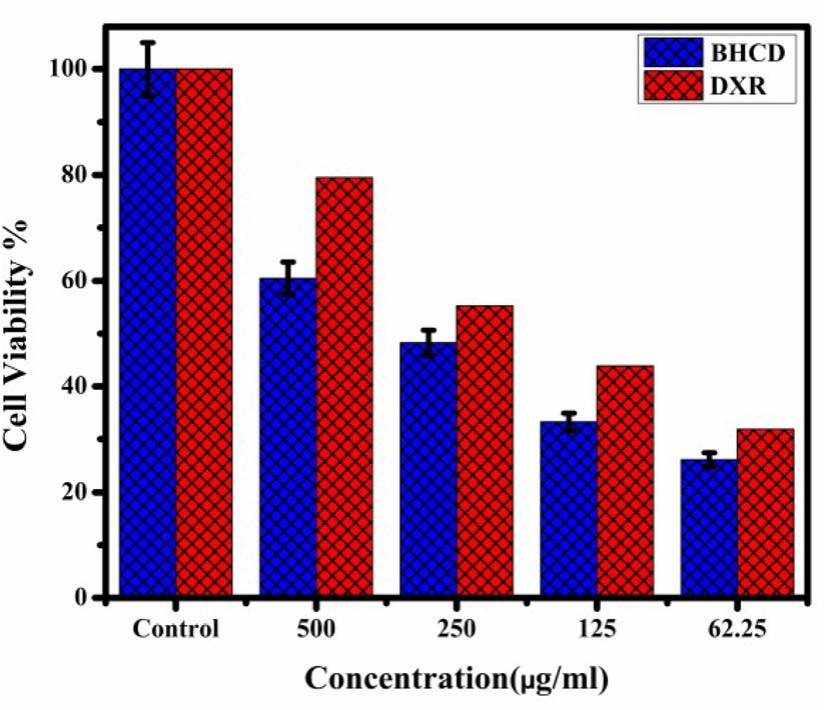
Cell viability graph of BHCD using MTT assay.

The cytotoxic effects of *Borreria hispida* derived carbon dots (BHCD) were studied using MCF7 (Michigan Cancer Foundation-7) cell lines, known to cause breast cancer in humans. For this study, BHCD concentrations ranging from 62.5 to 500 μg mL^−1^ were examined *via* the MTT assay to evaluate their impact on cell viability. Cytotoxicity tests were conducted on MCF-7 cells treated with BHCD at 62.5, 125, 250, and 500 μg mL^−1^ concentrations, as depicted in Fig. 16. At the maximum concentration of BHCD (500 μg mL^−1^), cell viability was observed to be 60%, as illustrated in Fig. 17.

Therefore, we suggest that BHCD exhibits a good level of cytotoxicity. The 60% cell viability result indicates that these carbon dots possess the potential for cytotoxicity against MCF7 cell lines.

**Fig. 18:**
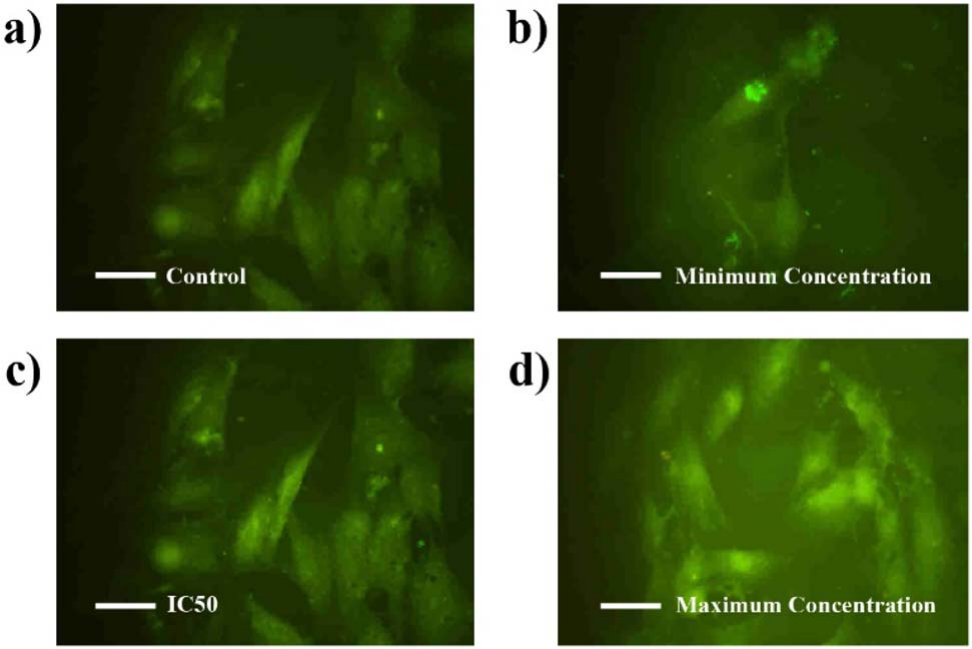
Confocal fluorescence images of MCF-7 living cells incubated with CDs (a) control. (b) Minimum concentration. (c) IC50. (d) Maximum concentrations.

Additionally, Fig. 18c and d display IC50 and maximum concentration of BHCD-treated MCF7 cells, showing a color change from dark green to light green as shown in Fig. 18. The IC50 concentration results in a partial color change, while the maximum concentration induces a complete color change compared to the control. The loss in membrane integrity and apoptotic induction, indicated by light green fragmented cells, is more pronounced in the maximum concentration group compared to the other treated cell lines exhibiting color changes in increasing order. The investigation confirms that BHCD-induced apoptosis is linked to ROS formation. From this assay, we conclude that increasing concentration correlates with an increasing color change, reflecting the cytotoxicity of BHCD against MCF7 cell lines.

## Conclusion

The synthesis of *Borreria hispida* carbon dots (BHCDs) from plant leaves has yielded remarkable nanomaterials with exceptional physicochemical properties. With an average particle size of merely 3.33 nm, high water solubility, remarkable photo-stability, and a substantial quantum yield of 40.8%, they demonstrate immense potential as a colorimetric probe for Fe^3+^ detection. The binding model, revealing a 2 : 1 ratio of BHCDs through a jobs plot, further accentuates their high selectivity and sensitivity, achieving an impressively low detection limit for Fe^3+^ ions. Validation using real water samples from natural environments such as ponds and lakes confirms the effectiveness of these BHCDs for practical environmental monitoring applications, positioning them as promising tools for sensitive and selective Fe^3+^ detection in real-world scenarios. The anticancer activity of BHCD against MCF7 breast cancer cell lines and the resulting 60% cell viability indicate a significant potential in biological applications.

## Conflicts of interest

The author declares no competing financial interest.

## Supplementary Material

RA-014-D4RA01686F-s001
