# Supplementary material for: Sustainable carbon dots from Borreria hispida: enhanced colorimetric sensing of Fe3+ ions and biological applications in live cell imaging
Source: RSC Adv. 2024 May 30;14(25):17471–9. doi: 10.1039/d4ra01686f (PMC11137498; doi:10.1039/d4ra01686f)
Supplement: RA-014-D4RA01686F-s001 [file RA-014-D4RA01686F-s001.pdf]

Electronic Supplementary Material (ESI) for RSC Advances.
This journal is © The Royal Society of Chemistry 2024

Sustainable Carbon Dots from *Borreria hispida*: Enhanced Colorimetric Sensing of Fe^{3+} Ions and Biological Applications in Live Cell Imaging

Shanmuga Priya S and Suseem SR*

Table S1: IC50 of BHCD

Concentration of Control Drug (µg/ml)				% of Cell Viability (Triplicate Values)			
	Trial 1	Trial 2	Trial 2	%Trial 1	%Trial 2	%Trial 3	Average
100	32.62195	31.9797	32.44898	67.37805	68.0203	67.55102	67.64979
50	38.61789	38.78173	39.18367	61.38211	61.21827	60.81633	61.13890333
25	52.03252	51.67513	52.2449	47.96748	48.32487	47.7551	48.01581667
12.5	67.27642	67.81726	67.2449	32.72358	32.18274	32.7551	32.55380667
6.25	75.4065	75.63452	75.61224	24.5935	24.36548	24.38776	24.44891333
Control	100	100	100	100	100	100	100

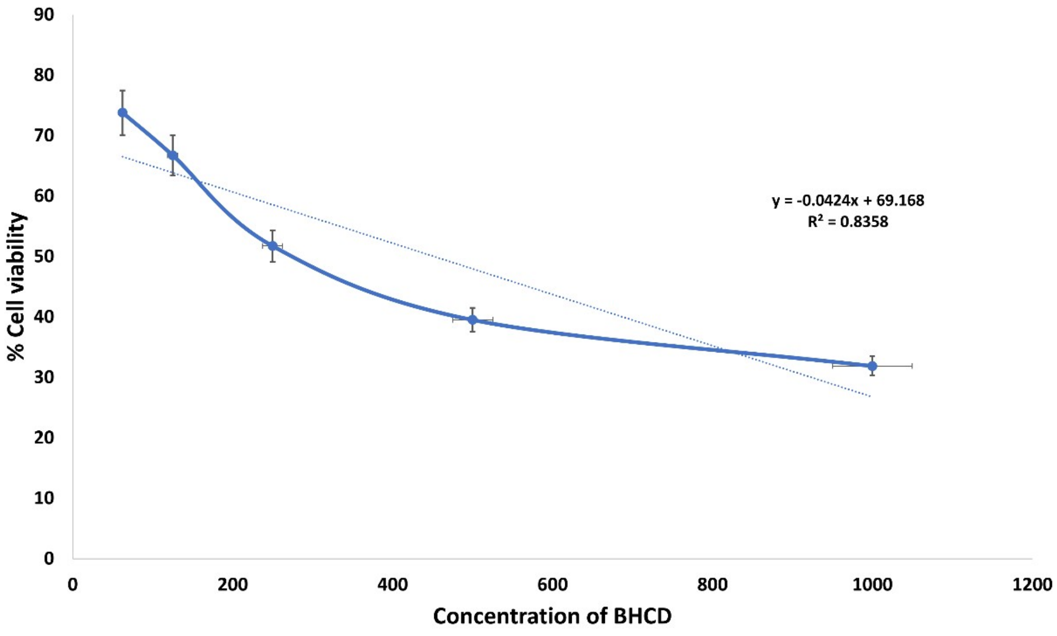

Fig. S1: Graph for Ic50 of BHCD

Table S2: Cell viability of standard drug

Concentration of Doxorubicin (µg/ml)				% of Cell Viability (Triplicate Values)			
	Trial 1	Trial 2	Trial 2	% t 1	% t 2	% t 3	Average
500	20.79832	20.50473	20.25316	79.20168	79.49527	79.74684	79.48126333
250	44.64286	44.26919	45.25316	55.35714	55.73081	54.74684	55.27826333
125	56.51261	55.94111	55.90717	43.48739	44.05889	44.09283	43.87970333
62.5	68.06723	67.50789	68.77637	31.93277	32.49211	31.22363	31.88283667
31.25	75.73529	74.02734	78.16456	24.26471	25.97266	21.83544	24.02427
Control	100	100	100	100	100	100	100

Table S3: Cell viability of standard drug

Concentration of BHCD (µg/ml)				% of Cell Viability (Triplicate Values)			
	Trial 1	Trial 2	Trial 2	% t 1	% t 2	% t 3	Average
1000	31.87817	32.14286	31.67006	68.12183	67.85714	68.32994	68.10297
500	39.08629	39.89796	39.61303	60.91371	60.10204	60.38697	60.46757333
250	50.86294	52.34694	52.03666	49.13706	47.65306	47.96334	48.25115333
125	66.80203	66.53061	66.90428	33.19797	33.46939	33.09572	33.25436
62.25	73.60406	74.28571	73.52342	26.39594	25.71429	26.47658	26.19560333
Control	100	100	100	100	100	100	100

Table. S4 . Comparison of this study with previously published articles

Name of the plant	Method of synthesis	size of carbon dot	Metal sensed	Limit of Detection	Reference
Borreria Hispida	Hydrothermal	3.33nm	Fe^{3+}	1.2 x10^{-6}	present work
canon ball fruit	Hydrothermal	11.2 nm	Fe^{3+}	0.071 µM	1
Tinospora cordifolia	Hydrothermal	5.47 nm	Fe^{3+}	0.414 µM	2
Sophora japonica	Hydrothermal	3.0 nm	Fe^{3+}	8.75 ppb (3σ/S)	3
flowers of wintersweet	Hydrothermal	1.5 nm	Cr (VI)& Fe^{3+}	0.07 µMand 0.15 µM,	4
Syringa obtata Lindl	Hydrothermal	2.76 nm	Fe^{3+}	0.11 µM	5
rose-heart radish	Hydrothermal	3.6 nm	Fe^{3+}	0.13µM (S/N = 3)	6
coriander leaves	Hydrothermal	2.387 nm	Fe^{3+}	0.4 µM	7
Polianthes tuberose L. Petals	Carbanization	4 to 6 nm	Fe^{2+} & Cu^{2+}	200 nM	8

Lycii Fructus	Refluxation	4 to 7 nm	Fe^{3+}	21 nM	9
cornstalk	Hydrothermal	5 .2 nm	Fe^{2+}	0.18 and 0.21 µM	43
onion waste	Hydrothermal	15nm	Fe^{2+}	0.31nm	44

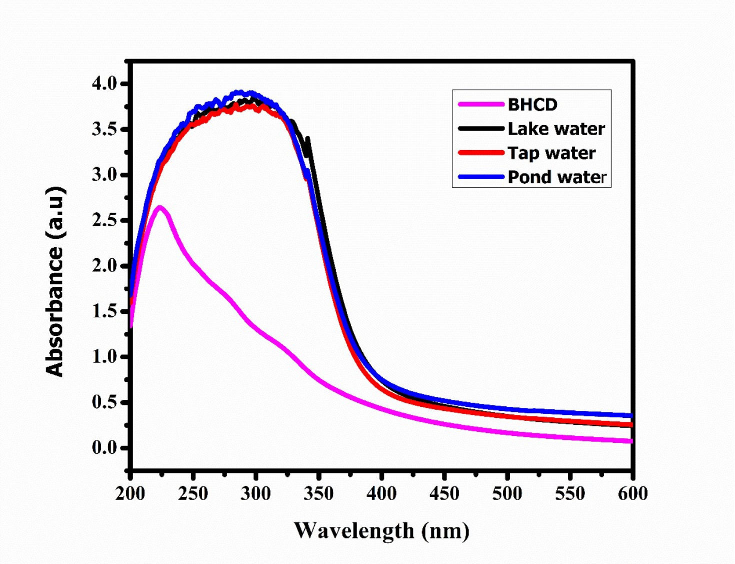

Fig. S2. UV-Vis spectrum of different water source with Fe^{3+}.

References:

1. Varman, G. A., Kalanidhi, K., & Nagaraaj, P. (2022). Green synthesis of fluorescent carbon dots from canon ballfruit for sensitive detection of Fe^{3+} and catalytic reduction of textile dyes. Dyes and Pigments, 199, 110101.
2. Mohapatra, D., Pratap, R., Pandey, V., Dubey, P. K., Agrawal, A. K., Parmar, A. S., & Sahu, A. N. (2021). Tinospora cordifolia Leaves Derived Carbon dots for Cancer Cell Bioimaging, Free radical Scavenging, and Fe 3+ Sensing Applications. Journal of Fluorescence, 1-18.
3. Hou, J., Wang, X., Lan, S., Zhang, C., Hou, C., He, Q., & Huo, D. (2020). A turn-on fluorescent sensor based on carbon dots from Sophora japonica leaves for the detection of glyphosate. Analytical Methods, 12(33), 4130-4138.
4. Xia, L., Li, X., Zhang, Y., Zhou, K., Yuan, L., Shi, R., ... & Fu, Q. (2022). Sustainable and green synthesis of waste-biomass-derived carbon dots for parallel and semi-quantitative visual detection of Cr (VI) and Fe^{3+}. Molecules,27(4), 1258.
5. Diao, H., Li, T., Zhang, R., Kang, Y., Liu, W., Cui, Y., ... & Sun, T. (2018). Facile and green synthesis of fluorescent carbon dots with tunable emission for sensors and cells imaging. Spectrochimica Acta Part A: Molecularand Biomolecular Spectroscopy, 200, 226-234.
6. Liu, W., Diao, H., Chang, H., Wang, H., Li, T., & Wei, W. (2017). Green synthesis of carbon dots from rose-heartradish and application for Fe^{3+} detection and cell imaging. Sensors and Actuators B: Chemical, 241, 190-198.
7. Sachdev, A., & Gopinath, P. (2015). Green synthesis of multifunctional carbon dots from coriander leaves and their potential application as antioxidants, sensors and bioimaging agents. Analyst, 140(12), 4260-4269.
8. Rooj, B., Dutta, A., Islam, S., & Mandal, U. (2018). Green synthesized carbon quantum dots from

polianthes tuberose L. Petals for Copper (II) and Iron (II) detection. Journal of fluorescence, 28, 1261-1267.
9. Sun, X., He, J., Yang, S., Zheng, M., Wang, Y., Ma, S., & Zheng, H. (2017). Green synthesis of carbon dots originated from Lycii Fructus for effective fluorescent sensing of ferric ion and multicolor cell imaging. Journal of Photochemistry and Photobiology B: Biology, 175, 219-225.
